# A hierarchy of needs for remote undergraduate medical education: lessons from the medical student experience

**DOI:** 10.1186/s12909-022-03479-4

**Published:** 2022-06-02

**Authors:** Henrike C. Besche, Sarah Onorato, Stephen Pelletier, Sepideh Ashrafzadeh, Ashwini Joshi, Brenna Nelsen, Jaewon Yoon, Joyce Zhou, Andrea Schwartz, Barbara A. Cockrill

**Affiliations:** 1 Office of Educational Quality Improvement, Harvard Medical School, TMEC 384 260 Longwood Avenue, Boston, MA 02115 USA; 2 New England Geriatrics Research Education and Clinical Center, Boston, MA USA; 3 U.S. Department of Veterans Affairs, Boston, MA USA

**Keywords:** Online teaching, Remote learning, Undergraduate medical education, Telehealth, Flipped classroom

## Abstract

**Purpose:**

The disruption of undergraduate medical education (UME) by the COVID-19 pandemic has sparked rapid, real-time adjustments by medical educators and students. While much is known about online teaching in general, little guidance is available to medical educators on how to adapt courses not originally designed for the online environment. To guide our faculty in this transition we conducted a needs assessment of students enrolled in virtual courses across all 4 years of UME training.

**Methods:**

Using a mixed-methods approach, we conducted a single-institution virtual learning needs assessment in May and June of 2020. We developed and disseminated a survey to assess student experiences with virtual learning. We conducted quantitative and qualitative analysis of responses (*n* = 255 or 39%) to identify emergent themes.

**Results:**

We identified six interdependent themes that need to be met for medical students to fully reach their learning potential: access to stable internet and quiet study spaces, flexible course design with asynchronous, self-paced components, clear expectations for engagement with content and each other, a sense of connectedness with faculty and peers, synchronous classes that maximize interactivity, and assessments that foster a sense of learning over performance. Interpersonal relationships with faculty and peers affected students’ sense of learning more than any other factor.

**Conclusions:**

Based on our findings we propose a hierarchy of needs for virtual learning that provides guidance on adapting existing medical school courses to the remote setting and overcoming common challenges. We highlight opportunities for how virtual elements may enrich in-person courses going forward, including in the clinical setting. Although the solutions required to meet the threshold of need at each level may differ based on the context, attending to these same fundamental needs can be extrapolated and applied to learners across a range of environments beyond the virtual.

**Supplementary Information:**

The online version contains supplementary material available at 10.1186/s12909-022-03479-4.

## Introduction

The COVID-19 pandemic catalyzed a rapid transition to a virtual learning environment for many undergraduate medical education (UME) activities. This shift impacted medical education in both pre-clerkship courses and clinical clerkships, necessitating that lectures, small group learning, clinical skills sessions, and assessments occur online [1, 2]. As a result, medical curricula adopted an increased use of asynchronous online learning, a greater engagement in telemedicine, and a shift to open-book testing [3, 4]. Reduction in clinical exposure, social isolation, lack of access to resources and technology, and difficulties engaging learners online have all been identified as common challenges with the rapid shift to remote learning in North America [5–7], and around the globe [8–11]. How these events will impact medical student learning is unknown.

Technology and asynchronous learning were already increasingly utilized by medical educators prior to COVID-19. A 2018 integrative review found that barriers to online medical education included inadequate technology infrastructure, lack of time and institutional support, increased cognitive load, and negative attitudes of educators towards virtual learning in the US [12]. Furthermore, best practice for online learning suggest that educators avoid simply reproducing in-person instruction virtually and instead, optimize technology for content delivery, interaction, and innovative assessment [13]. Despite some evidence that online instruction can be effective in UME, many questions about design principles for digital learning remain [14]. In addition, students’ disconnection from other students and faculty has been described as a significant barrier to virtual learning [15, 16]. Similarly, in clinical settings, online instruction has been found to complement—but not replace—experiential learning; although students report satisfaction with aspects of online modalities, a significant proportion want further clinical interaction [17, 18].

We anticipate that even when in-person activities can fully resume safely, there will be an increased role for technology and telemedicine in medical education [16, 19, 20], requiring careful negotiation of strengths and limitations in hybrid learning [8, 21, 22]. Given the paucity of evidence-based literature on adapting medical curricula to the remote setting, we surveyed students across all 4 years of our UME curriculum about their experience with virtual learning in order to provide guidance to faculty. Using a mixed-methods approach, we identified six interdependent themes and propose a hierarchy of needs that needs to be met for successful virtual learning.

## Methods

This study was developed in collaboration with the Harvard Medical School (HMS) Office of Educational Quality Improvement and the HMS Academy. The HMS Educational Research Committee deemed the survey not human subjects research and therefore not subject to further HMS Institutional Review Board review.

### Survey development

We developed a 34-question survey including up to eight free-response questions (Additional file 1). Other questions were multiple-choice items scored on a 5-point Likert scale. The survey was developed on the Qualtrics survey platform and employed branching logic to target specific questions towards relevant groups of learners based on self-reported course enrollment ranging from small clinical electives to class-size first year courses. Topics included in the survey were identified through a combination of focus groups and informal conversations with students and faculty. Ultimately, areas queried included best practices for use of technology, class-time allocation, asynchronous learning, assessment, relationships with peers and faculty, barriers to virtual learning, clinical skill development, and clinical experience in preparation for residency applications.

### Survey distribution

Our study was a single-center needs assessment of medical students. The survey was sent via email to all 654 medical students across all class years and learning settings, including pre-clerkship courses (including a longitudinal clinical skills course), clinical clerkships, and post-clerkship courses and clinical electives. Two hundred fifty-five students responded, a response rate of 39%. Students completed the survey in May and June of 2020.

### Qualitative analysis

A qualitative analysis was conducted for the eight free-response questions. Each question was reviewed and coded independently by two medical student reviewers (BN, JY, JZ, SA, SO). An iterative, inductive approach was undertaken to code the qualitative data. Our thematic analysis was guided by a modified constructivist grounded theory (CGT) approach, a method that is useful in understanding complex phenomena that are not adequately described by existing models [23]. Furthermore, a CGT approach allowed for the construction of theories to describe observed attitudes and behaviors as we developed and evolved our understanding of emergent themes.

Given the overlap in question content across three of the free response questions, one set of codes was developed for these three questions. The other five individual free response questions were coded individually, with discrete codes developed for each of the items. For all of the survey items, coding was conducted in an iterative manner, with reviewers individually identifying themes, discussing differences, and revising the codes for each response.

An independent medical student reviewer (AJ) who was not involved in the initial data codebook development or analysis subsequently collated codes from all items and organized them into overarching thematic categories. This approach allowed for unbiased analysis of the qualitative data, and themes were subsequently reviewed and agreed upon by all reviewers. The data were organized according to these larger themes. Representative quotes were selected within each individual code and considered for each overarching thematic category.

### Quantitative analysis

Descriptive statistics were generated for all survey questions. Where appropriate, Likert response options were collapsed into categories and analyzed using the Chi-Square Test of Independence. In the few instances where means are analyzed, Independent Samples t-Tests were employed. Quantitative analysis was conducted using IBM SPSS Statistics for Windows, Version 27.0 (Armonk NY:IBM Corp).

## Results

Data were gathered in May of 2020, roughly 2 months into the rapid transition to teaching remotely. All students across the curriculum were enrolled in virtual courses ranging from classroom-based courses in the pre- and post-clinical curriculum to clerkships and electives. The qualitative data analysis process yielded 65 unique codes from across the eight free response survey items, which were organized into six themes (Table 1). Below we present each theme with representative quotations and supporting quantitative data. The order of presentation is informed by the logical connection between themes, which in a secondary and final step of the analysis led us to propose a hierarchy of needs for virtual learning that we will explore in the discussion.

**Table 1:** Summary of qualitative analysis. Abbreviated list of themes and codes (for a complete list of all codes see Additional file 2). Each theme will be further explored in the results in the order presented here. The order in which themes are presented was chosen to best allow integration with quantitative data

	Themes	Sample of Included Codes
**1**	Role of technology and virtual resources for enhancement of learning	• Virtual experience • Technology • Resources
**2**	Flexibility	• Asynchronous learning • Time efficiency • Self-directed learning
**3**	Effects of learning in an exclusively virtual environment	• Learning • Alternative modalities • Length of class time
**4**	Sense of connection to people and course material through interpersonal interactions	• Engagement • Participation • Maintaining relationships
**5**	Professionalism in the virtual environment	• Accountability • Etiquette • Expectations
**6**	Adaptation of assessment and feedback to virtual environment	• Assessments • Presentations • Faculty feedback

### Role of technology and virtual resources for enhancement of learning

The most basic theme that emerged centered around technology. Unstable internet connection often or always affected 20% (*n* = 36) of all respondents, and 18% (*n* = 32) often or always struggled to find a quiet space [24]. Among all respondents, 43% (*n* = 76) reported experiencing issues with *both* internet connection and finding quiet space at least sometimes: “In general, lower than optimized internet connection and background noises are things I continue to struggle with but I don’t think it’s something [our institution] can control. Working with people from different time zones and different commitments has been challenging.”

**Fig. 1:**
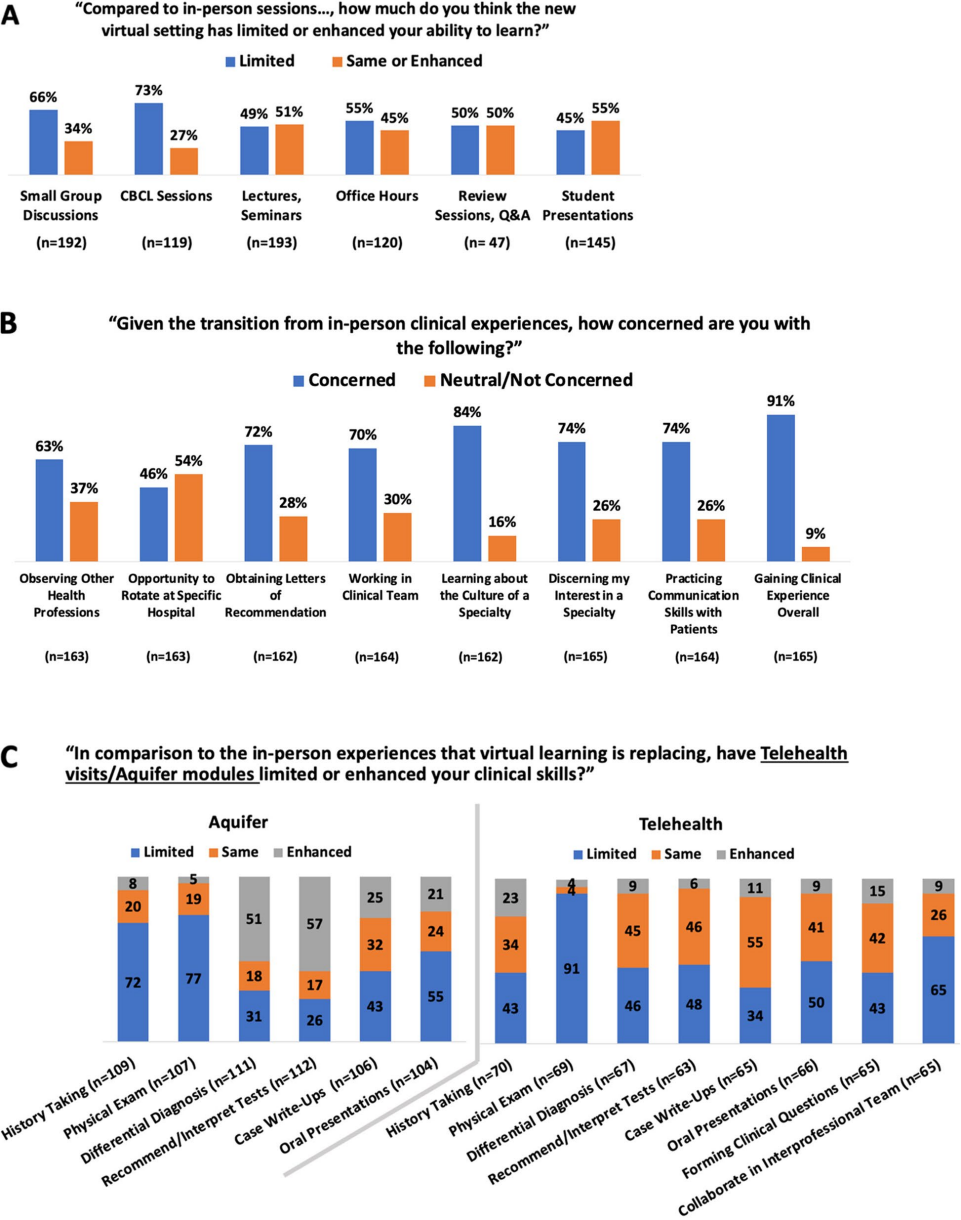
Effect of the remote experience on the perception of learning. Likert response options were collapsed into categories to allow for easier comparison. **A** Discussion-based formats (small groups or case-based collaborative learning (CBCL) were harder to translate to the remote setting compared to lectures or other formats. Overall, at least half of all students felt that the remote setting limited their learning compared to the in-person classroom experience. **B** In the clinical setting students were concerned about their learning across many different domains. When probed more specifically, **C** Aquifer cases, and **D** Telehealth visits provided opportunities for students to enhance the clinical skills in some domains

When asked how virtual learning compared to the in-person equivalents, a majority of students reported that the virtual setting limited their ability to learn across a variety of formats (Fig. 1A). Students that struggled with unstable internet connection at least sometimes found learning particularly limiting during lectures/seminars (chi square *p*-value < 0.035), and student-led presentations (chi square *p*-value < 0.026): “In instances where I struggled with internet connectivity, I would miss out on key points that I felt awkward asking for a clarification about. It is easier to lose engagement and get distracted when learning virtually from home, especially in class session[s] that did not facilitate active participation.” Lack of access to quiet spaces mostly limited learning from student-led presentations (chi square *p*-value < 0.067) but no other format. Notably, the 43% of students that reported struggling with both – access to quiet space *and* stable internet - did *no*t report feeling significantly more limited in their learning than others (Additional file 3).

Clinical teaching was interrupted by COVID-19 as well, and 87% (*n* = 144) of students enrolled in virtual clinical courses were concerned about their learning in general and across several domains (Fig. 1B). Students who had already participated in telehealth visits (51%, *n* = 82) found that this mode of clinical engagement limited learning in many domains such as physical examinations or working on healthcare teams compared to in-person, yet it also enhanced learning in several areas, including history taking, forming clinical questions/retrieving evidence, and case write ups (Fig. 1C): “Because the medical knowledge base is even more so at our fingertips than during in-person clinical patient encounters and rotations, I think that virtual learning provides an opportunity to enhance differential diagnosis and test interpretation skills, to further review and consolidate knowledge, and to examine existing literature to answer clinical questions. Additionally, calling patients to get a history actually makes the history taking more focused, efficient, and complete.”

Aquifer cases (Aquifer, Lebanon, NH, USA) allow for asynchronous engagement with clinical cases and were used by 71% (*n* = 113) of respondents participating in virtual clinical courses. Despite similar limitations described with telehealth visits, students reported advantages in virtually learning about case-write ups, differential diagnosis formation, and recommending/interpreting tests compared to in-person clinical experiences (Fig. 1D): “The transition to clinical training may be less overwhelming for students in this setting and allow for more feedback, role playing, and discussion of differential diagnoses.”

### Flexibility

When asked to envision an ideal virtual environment, students highlighted flexibility as critical for success: “Ideally, I would have flexibility in how I plan my studying. I would also appreciate a well-structured curriculum that highlights key concepts, in addition to having additional work that I could use to enhance my learning.” Asynchronous activities were important in this context: “There are advantages that come with online learning, like being able to record sessions and engage in more self-paced learning, and I think that it is good to use those advantages to give more flexibility to those students who need it during this time.” Learning from experts outside the institution was another recurring theme: “A major advantage of online learning is that it offers us greater opportunity to learn from folks outside [of town] who otherwise would not be able to teach us in-person.” Yet, students cautioned that lesson plans needed to be carefully planned and executed: “There seems to be a lot of killing time out of obligation, i.e., the students paid to learn so we will give them a lecture/assignment, but the lesson is poorly constructed and the work feels like busy work.”

Despite significant concerns about virtual clinical learning discussed above, students also identified opportunities due to fewer logistical restraints: “I think [virtual learning] provides the opportunity to attend a wide variety of clinics (if they support telehealth), even in the same day, which would be logistically difficult otherwise. I am getting a lot of exposure that I would not have otherwise.” “[Virtual learning affords] opportunities to seamlessly transition from one service/team/session to another given that everything is virtual. Students can now ‘be in the OR’ and then debrief with residents/attendings simultaneously or just moments after by switching Zoom calls.”

### Effects of learning in an exclusively virtual environment

**Fig. 2:**
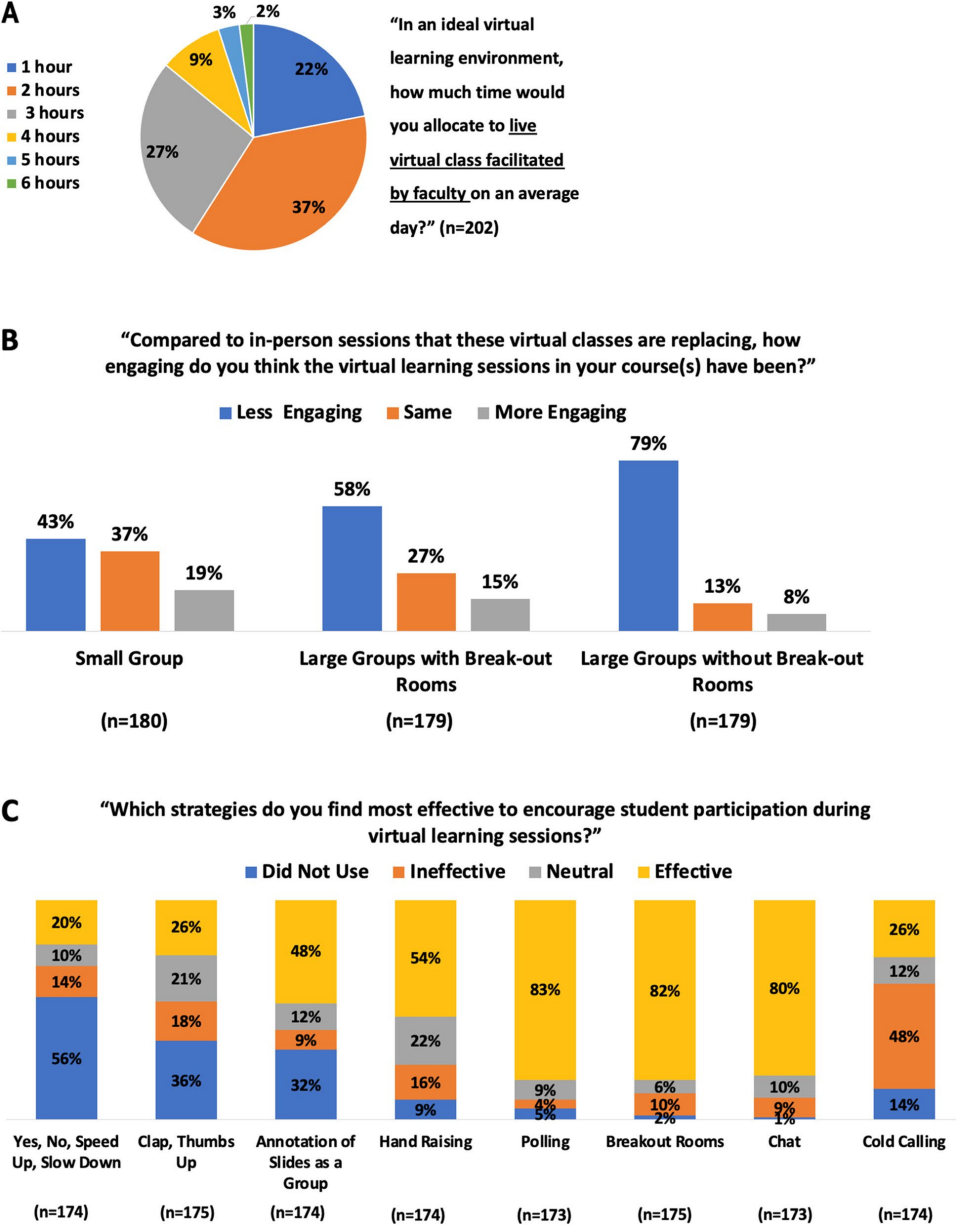
Engaging learners virtually. Likert response options were collapsed into categories to allow for easier comparison as needed. **A** Zoom fatigue put a limit on the learners’ attention and engagement. Students indicated that no more than 3 hours of live virtual class per day was ideal. **B** Overall virtual learning was seen as less engaging than in-person. Small groups or formats including breakout-groups were rated as more engaging. **C** Breakout rooms, polling and chat were rated as most effective tools in engaging students virtually

Synchronous online meetings lead to cognitive overload also known as Zoom fatigue [25]. Students commented on Zoom fatigue as a barrier to virtual learning, with 86% (*n* = 174) of students preferring total live class time be limited to 3 h or fewer per day (Fig. 2A): “The biggest thing I have learned, is that [synchronous] virtual lectures are very, very exhausting. For some reason it is very taxing to watch the virtual lectures, more so than in person lectures […]. I also think it is even more important with virtual learning to allocate ample time to individual learning and consolidation.” Preference for time allocation was independent of whether students experienced issues with connectivity or space (Additional file 3).

The flipped classroom model [26, 27] was frequently suggested as a strategy to optimize synchronous online class time: “[The best virtual sessions] assigned preparatory material. [They] use session time to practice applying concepts with some review. “[They made] efficient use of time and limited the hours of screen time.”

Overall, virtual lessons were rated as less engaging than the in-person experience (Fig. 2B): “It is [disappointing] to see students keep their [camera’s off] and not participate, when I know that in person they would be actively participating. Something about Zoom gets rid of that normal accountability.” Breakout rooms, polling, chat and annotations were rated as the most effective tools to promote engagement in live classes (Fig. 2C): “Chat feature and breakout rooms [can be] used well to facilitate question-asking and small group discussion.” Polling also facilitated immediate feedback on whether the students were effectively learning: “I think that some of the biggest opportunities for online learning are the real-time (potentially anonymous) feedback that a professor can gather from the class and use this information to clear up any confusion or re-emphasize key concepts.”

### Sense of connection to people and course material

Of all the factors that we analyzed, interpersonal relationships stood out as having the biggest impact on learning. After classroom-based courses transitioned online, 49% of students experienced relationships with *both*, faculty and peers, more negatively. Importantly, students who perceived more negative relationships with faculty or peers felt less comfortable participating in class and had a diminished sense of learning (Table 2): “I felt like I didn’t know my faculty. It felt hard to stop the class to ask a question if I didn’t understand something.”

**Table 2:** Effect of relationships on participation and ability to learn. After moving online, 59% (*n* = 106) of respondents rated their relationships with peers more negative. 55% (*n* = 99) reported relationships with faculty as more negative. Nearly half of respondents (49%) reported their relationships with *both* faculty and peers as more negative. This group is most likely to say that virtual learning has limited their ability to learn and less comfortable participating in class. Problems with internet or access to quiet space did not seem to affect relationships (Additional file 3). Likert response options were collapsed into categories and analyzed using the Chi-Square Test of Independence

	Relationship with Faculty and Peers more Negative (49% *n* = 79)	Other (51% *n* = 81)	Chi-Square *p*-Value
**% Limited My Ability to Learn**			
Small group discussion (*n* = 154)	77	58	.011
CBCL sessions (*n* = 92)	76	71	.600
Lectures/Seminars (*n* = 157)	61	38	.003
Office hours (*n* = 102)	69	40	.003
Review sessions (*n* = 121)	59	40	.036
Student presentations (*n* = 121)	65	27	<.001
**% Less Comfortable Participating**			
Ask a question (*n* = 158)	70	43	<.001
Answer a question (*n* = 158)	68	39	<.001
Challenge ideas (*n* = 152)	73	44	<.001
Make a presentation (*n* = 136)	45	11	<.001

The lack of informal classroom interactions emerged as a potential driving force for this trend in student-faculty interactions: “I feel that I don’t really know my faculty the way that I would in an in-person setting. The casual interactions that happen after class when you go up to ask a question just can’t really happen virtually in an organic way.” This concern also applied to peer-to-peer interactions: “I am used to becoming more close to my peers because we often talk before or after small group sessions in-person. Online, we don’t talk with each other because initiating conversations with peers you have not met before is a bit awkward.”

Breakout rooms stood also out as potential opportunity for students to connect: “Breakout rooms with small numbers of people are good…a few minutes should be built in for socializing because we’re all isolated.” Notably, case-based collaborative learning [28], a format that combines frequent breakout groups with large group discussion, was the only format in which the respondents’ sense of learning was the *not* affected by negative relationships (Table 2), indicating that virtual classes can work well: “An ideal virtual class is in a small group setting with peers and a faculty member. I think this is the best way to facilitate both discussion, creative thinking between peers, and real-time clarification with faculty.”

### Professionalism in the virtual environment

Survey respondents discussed the need to set expectations for professional behavior among students; specific areas that students commented on as important for expectation setting largely reflected the different challenges students experienced with the virtual environment. These areas included social norms in how to use technology: “Show up on time, mute when in large groups, work in quiet space free of distractions when possible, camera on when able (especially in smaller groups).” Others appealed for students to engage and be accountable: “Try to hold yourself to the same standards that you would in-person—e.g., come to class prepared, refrain from using devices for things other than cases while the group is meeting.”

Some students also found unexpected opportunities in developing professional skills, such as communicating in groups: “The biggest benefit I see in virtual experiences for clinical training is that, because of the artificial interface, it is much harder for faculty/residents and even peers to interrupt students as they are speaking. It has forced residents to let students reason out loud without cutting them off. It has forced faculty listen to an entire oral presentation without interruption. In this way I think that students are actually being pushed to ‘put their nickel down’ and verbally work through clinical reasoning more thoroughly than I ever had to on the wards in person.” Another student remarked: “Since I’m soft-spoken and have trouble projecting my voice, I actually really appreciated being able to ask questions with a mic[rophone or via] chat box.”

For some students the virtual setting also promoted greater support for personal and career development: “On my virtual [sub-internship] I think I received more one-on-one time with my attendings, which allowed me to incorporate feedback and grow throughout the elective. In addition, I felt comfortable asking for advice on choosing specialties and applying to residency.”

### Adaptation of assessment and feedback to virtual environment

Remote learning poses particular challenges for assessment and at least temporarily led to more open-book testing [3]. Students described that they felt faculty perceptions of students were more heavily influenced by assessments: “I think for me personally I feel a lot more pressure to do well on tests in the virtual environment because I feel like it’s the only metric faculty have to judge me by.” This challenge, along with concerns about the integrity of virtual exams, led to recommendations for other ways of tracking progress: “There needs to be something more like individual projects/activities that students do and then present or more quizzes/assignments to give the faculty something to assess. With the current [virtual learning] platform as far as I see it people can really only be assessed fairly by quizzes/tests (but even this is tainted, because it is impossible to ensure that students do not use outside resources).”

Yet, students cautioned that excessively stringent rules around assessment would undermine learning and well-being and strongly emphasized the importance of faculty and course leaders conveying trust in learners: “Threatening students with rules and proctoring will only make this experience more difficult. It will feel like administration does not trust us, which is not what we need to be thinking about when learning from home.” Students also discussed setting clear expectations with regard to assessment as a tool for learning, as opposed to evaluation, as helpful to encourage students to act with integrity during online assessments: “I think emphasizing that the [test] modules are primarily for learning, not evaluation, helps keep stress low and encourages honest answers to questions (i.e., without looking things up, primarily as a knowledge assessment).”

## Discussion

Our virtual learning survey was designed as a needs assessment, with the goal to support our institution’s medical educators and students at a time of rapid change. We set out to understand both challenges and opportunities associated with the virtual setting, *not* to provide an assessment of whether in-person teaching or virtual teaching was better than the other.

**Fig. 3:**
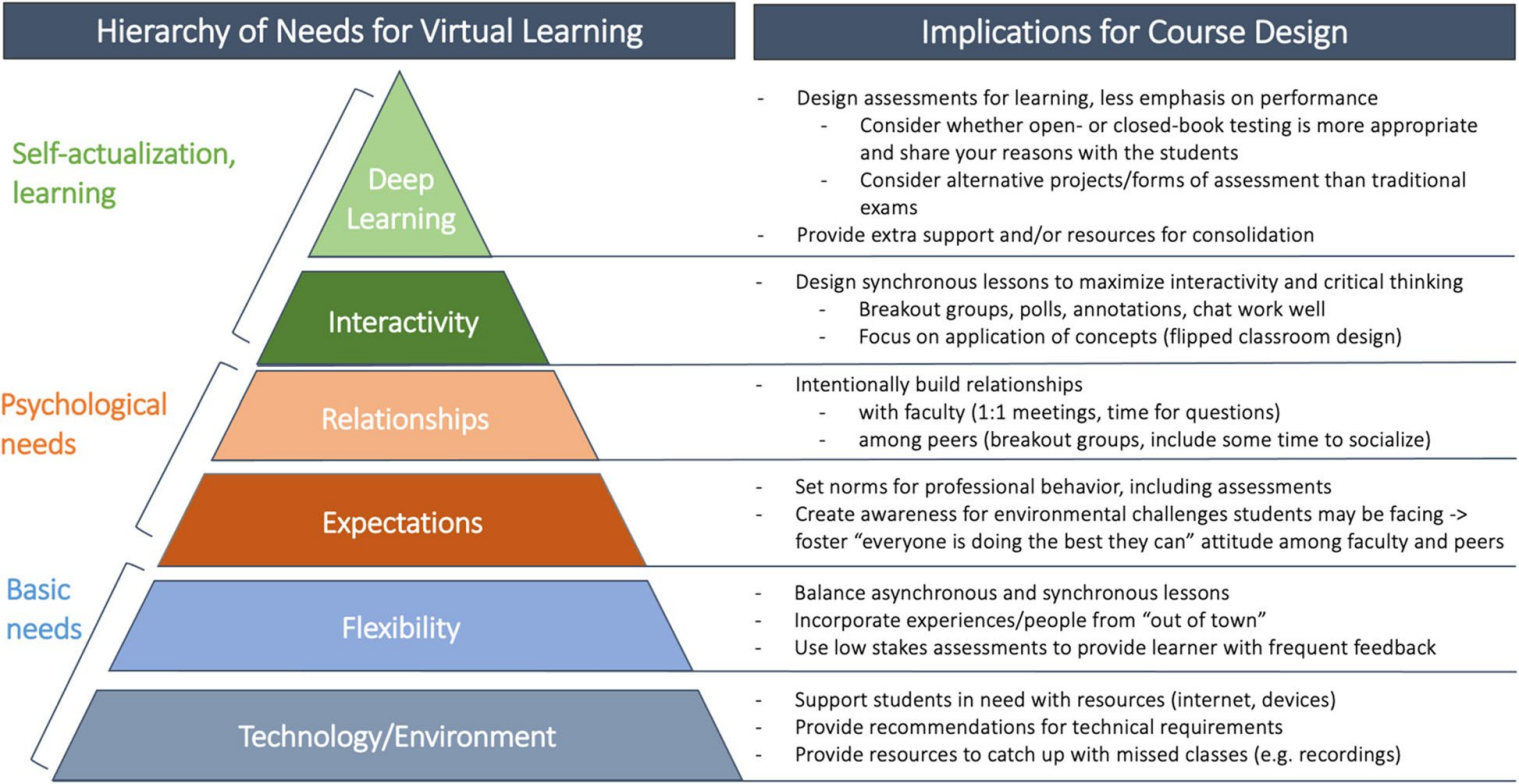
Schematic model for a hierarchy of needs for virtual learning and implications for course design based on Maslow’s hierarchy of needs. This model emerged organically when we synthesized qualitative and quantitative findings into one narrative. Each level of the pyramid needs to be fulfilled to a certain threshold, e.g. internet that is table most of the time, to be able to benefit from the next level of the pyramid. Course design can ensure that each of these thresholds is met for as many students as possible

After comparing the qualitative and quantitative data and exploring interdependencies (Table 2 and Additional file 3), we concluded that a successful virtual learning environment requires satisfying stepwise, interdependent thresholds (Fig. 3). This is similar to Maslow’s model for self-actualization, in which needs at lower levels of the hierarchy must be met in order to facilitate attainment of higher levels [29].

### Technology and environmental factors

Access to quiet space, adequate internet speeds, and proper technology have been identified as environmental barriers to online learning in medical schools around the world, before and during the pandemic [7–11]. The degree to which medical schools may be able to support these learners by providing additional resources may vary. It is critical that faculty design courses in ways that anticipate some challenges with access to these basic needs, and meet these challenges with understanding and support, including possibilities to catch up with work asynchronously (see flexibility).

### Flexibility

Students in our and other studies perceived the inherent flexibility in virtual learning as an advantage [7, 11]. In addition, bringing in experts from beyond the local campus was greatly appreciated, and warrants consideration when in-person learning resumes.

While greater flexibility was overall seen as advantage, it required careful instructional design. Students noted that the remote setting can also foster confusion, and a perceived lack of supervision over learning particularly for asynchronously delivered content. Well-structured assignments paired with frequent low-stakes assessment helped students to make the most of asynchronous learning. While screen fatigue needed to be avoided, students appreciated access to some synchronous classes as opportunity to clarify, synthesize, and critically assess their understanding with faculty and peers.

### Expectations

Virtual learning requires additional class norms around virtual etiquette (e.g., use of video camera or muting of microphone during group sessions) and unobserved assessments (e.g., clear instructions about which outside resources are acceptable during exams). Many students highlighted the importance of self-motivation and assumption of integrity as guiding principles for faculty when formulating and communicating expectations for virtual courses.

Empathy and striking a non-judgmental tone plays an important role when setting expectations in the virtual environment. Many students and faculty might not be aware of the unique challenges that learners may be facing, especially during the pandemic, such as difficulties with technology and the physical environment as well as coping with financial, mental health and/or other stressors. In addition to addressing these concerns through actions at lower levels of the hierarchy, educators should promote the basic assumption that all class members are engaging with the course to the best of their abilities within the constraints of their particular situation [24].

### Relationships

The importance of investment in relationships in the virtual learning environment cannot be overstated. Of all the aspects we looked into, we found that a lack of meaningful relationships with both faculty and peers was associated with much greater reported limitation of learning than any other factor, including internet connection and quiet study space (Additional file 3). Thus, while fostering student-student and student-faculty relationships and creating opportunities for informal interaction takes more effort virtually than in-person, finding opportunities to build these relationships remains a crucial need. Self-determination theory recognizes “relatedness” - the sense of belonging to a community - as a critical factor in promoting self-regulation and learning [30]. Our data support this theory and suggest that medical students cannot achieve learning to their highest potential without authentic relationships and engagement with each other.

### Interactivity

Students struggled to stay engaged during virtual class much more than in-person. Educators should be mindful of incorporating frequent interactive experiences in order to maximize student learning and engagement. For example, polling, slide annotation, and chat functions are readily available on most online learning platforms and can substantially lower the barrier to student participation. Breakout rooms and other opportunities for small group learning were critical to foster participation, as well as peer-peer relationships. In addition, learning activities during synchronous sessions should be focused on higher-order thinking through emphasis on application, analysis, and evaluation [31].

### Deep learning, assessment and professionalism

Virtual learning raises questions about feasibility and utility of closed book testing [3]. We found that the virtual setting reduced learners’ belief in the fairness and purpose of examinations. Faculty are encouraged to set transparent operational norms around assessment, and to reinforce the broader expectation that assessments serve the purposes of learning, feedback, and self-reflection as opposed to the evaluation and comparison of student performance.

Although many medical schools already use pass-fail grading criteria for assessment, explicitly going over the goals and objectives of assessments in the virtual environment may reassure students in order to better support students’ learning, formative evaluation, and professional development.

### Clinical education in the virtual environment

While our hierarchy of needs focuses on the classroom setting, our results also identified many challenges with virtual clinical learning, supporting the notion that clinical education is best conducted in person. However, students identified a number of opportunities for blended learning that may enrich their education. Over half of students felt that history taking, creating clinical questions, retrieving evidence, creating a differential diagnosis, interpreting tests, and case writeups were *not* limited through telemedicine compared to in-person patient interactions. Validated telemedicine physical exam tools and clinical education strategies are growing in usage and have been shown to be effective [32], especially as telemedicine becomes increasingly integrated into our healthcare delivery system [33–36]. We conclude that while some aspects of data gathering cannot be adequately replicated virtually, consolidation of knowledge can be enhanced through asynchronous modules and/or cases [33]. Furthermore, asynchronous learning can standardize the breadth of cases that students experience during their clinical rotations, as is done already at our institution. More work is needed to study possibilities to enrich in-person clinical teaching with hybrid experiences in the future.

## Conclusion

Teaching through this pandemic has been both challenging and transformative for many educators. The rapid advent of virtual teaching is likely to impact higher education for years to come with increasing utilization of virtual or hybrid approaches to supplement traditional in-person course offerings. The hierarchy of needs for virtual learning can provide guidance for educators developing virtual, hybrid or in-person courses going forward. While the specific solutions to satisfy each step of the pyramid may vary depending on the setting, we believe that the general framework sheds new light on how different elements of course and instructional design influence students’ ability to learn.

## Limitations

This study was conducted at a single site and thus results may be limited in their generalizability. The survey data are subjective to both nonresponse bias (students who did not respond could differ and hold opinions different from those who did) as well as response bias (students respond inaccurately due to a variety of subconscious or conscious influences). In addition, the free-response items were optional and there is an inherent subjectivity to qualitative analysis. Data were gathered in May 2020 and present a certain moment in time; medical students’ attitudes may have changed since then. In addition, at that time the only tool used to conduct synchronous lessons was Zoom. Results may differ using different software. Despite these limitations, this survey presents important insights into medical student responses to the shift to virtual learning and can inform student-centered online medical education during the pandemic and beyond.

## Supplementary Information

**Additional file 1.**

**Additional file 2.**

**Additional file 3.**

**Additional file 4.**

## Data Availability

The data that support the findings of this study are available from the corresponding author upon request.

## Abbreviations

UME: Undergraduate medical education
HMS: Harvard Medical School
CGT: Constructivist grounded theory
