# Supplementary material for: A hierarchy of needs for remote undergraduate medical education: lessons from the medical student experience
Source: BMC Med Educ. 2022 Jun 2;22:423. doi: 10.1186/s12909-022-03479-4 (PMC9161626; doi:10.1186/s12909-022-03479-4)
Supplement: Supplementary file 1 — Additional file 1. [file 12909_2022_3479_MOESM1_ESM.pdf]

Virtual Learning Survey - May 2020

Start of Block: Default Question Block

Q51 **This 10-minute quality improvement survey is intended to assess your experiences with virtual learning at HMS in order to improve current and future virtual learning experiences. All results of this survey will be de-identified prior to analysis.**

Q4 **Background Questions**

Q55 Which program are you enrolled in?

- ○ Pathways Medical (1)
- ○ HST (2)
- ○ Pathways Dental (3)

Q2 In which phase of the curriculum are you currently enrolled?

- ○ Pre-clerkship (1)
- ○ PCE (2)
- ○ Post-PCE student (3)
- ○ Graduating student (4)

Q3 What courses have you recently taken part in that utilized virtual learning?

- ▢ Pathways pre-clerkship courses (1)
- ▢ HST pre-clerkship courses (2)
- ▢ Virtual Radiology course (3)
- ▢ Essentials of the Profession II (4)
- ▢ Clinical Capstone (5)
- ▢ AISC (6)
- ▢ COVID Curriculum Elective (7)
- ▢ Virtual Clinical Elective (8)
- ▢ Clerkship-specific Online Modules (10)
- ▢ Other (9) ____

Q41 **Thinking about your recent experiences with virtual learning at HMS ...**

Q52 In an **ideal virtual learning environment,** how much time would you allocate to the following activities **on an average day?**

	1 hour (1)	2 hours (2)	3 hours (3)	4 hours (4)	5 hours (5)	6 hours (6)	7 or more hours (7)
Live virtual class facilitated by faculty (1)	○	○	○	○	○	○	○
Working remotely by yourself (2)	○	○	○	○	○	○	○
Collaborating remotely with peers (4)	○	○	○	○	○	○	○

Q54 Imagine an **ideal virtual class**, what would it look like? Where do you see the biggest opportunities for online learning?

____

____

____

____

____

Q16 Compared to in-person sessions that live virtual classes are replacing, how much do you think the new virtual setting has **limited or enhanced** your ability to learn?

	Not applicable (6)	Severely limited (1)	Somewhat limited (2)	Same (3)	Somewhat enhanced (4)	Greatly enhanced (5)
Small group discussions (1)	○	○	○	○	○	○
CBCL sessions (2)	○	○	○	○	○	○
Lectures, seminars (3)	○	○	○	○	○	○
Office hours (4)	○	○	○	○	○	○
Student presentations (5)	○	○	○	○	○	○
Review Sessions, Q&A (7)	○	○	○	○	○	○
Journal Club (8)	○	○	○	○	○	○
Other (6)	○	○	○	○	○	○

Q25 Recall virtual classes that went well. How did they enhance your learning?

____

____

____

____

____

Q26 Recall virtual classes that did not go as well. How did they limit your learning?

____

____

____

____

____

Q58 Compared to in-person sessions that live virtual classes are replacing, how much do you think the new virtual setting has limited or enhance faculty's ability to...

	Not Applicable (1)	Severely limited (2)	Somewhat limited (3)	Same (4)	Somewhat enhanced (5)	Greatly enhanced (6)
Assess your medical knowledge (1)	o	o	o	o	o	o
Observe you directly (2)	o	o	o	o	o	o
Provide feedback (3)	o	o	o	o	o	o
Assess your degree of professionalism (4)	o	o	o	o	o	o

Q20 In what way has the transition to virtual learning influenced your relationships with...

	Much more negative (1)	Somewhat more negative (2)	Same (3)	Somewhat more positive (4)	Much more positive (5)
Faculty (1)	○	○	○	○	○
Peers (2)	○	○	○	○	○

Q21 In what way(s) did your relationships change?

____

____

____

____

____

Q13 Compared to in-person sessions that these virtual classes are replacing, how engaging do you think the virtual learning sessions in your course(s) have been?

	Much less engaging (1)	Less engaging (2)	Same (3)	More engaging (4)	Much more engaging (5)
Small group setting (1)	○	○	○	○	○
Large-groups with break-out rooms (2)	○	○	○	○	○
Large-groups without break-out rooms (3)	○	○	○	○	○

Q10 Compared to in-person sessions that these virtual classes are replacing, how comfortable are you doing the following?

	Did not use (6)	Much less comfortable (1)	Less comfortable (2)	Same (3)	Somewhat more comfortable (4)	Much more comfortable (7)
Asking a question verbally (1)	○	○	○	○	○	○
Answering a question verbally (2)	○	○	○	○	○	○
Giving a presentation (3)	○	○	○	○	○	○
Challenging ideas (4)	○	○	○	○	○	○

Q27 **Thinking specifically about your clinical virtual sessions (e.g. POM, Capstone)**

Display This Question:

If In which phase of the curriculum are you currently enrolled? = Pre-clerkship

Q28 Thinking about your virtual clinical sessions, how do you think these sessions have **limited or enhanced** development of your clinical skills.

	Not Applicable (6)	Severely limited (1)	Somewhat limited (2)	Same (3)	Somewhat enhanced (4)	Greatly enhanced (5)
History-taking (1)	○	○	○	○	○	○
Physical examination (2)	○	○	○	○	○	○
Differential diagnosis (3)	○	○	○	○	○	○
Case write-ups (4)	○	○	○	○	○	○
Oral presentation (5)	○	○	○	○	○	○

Display This Question:

If In which phase of the curriculum are you currently enrolled? = PCE

Q29 Thinking about your virtual clinical sessions, how do you think these sessions have **limited or enhanced** development of your clinical skills.

	Not Applicable (6)	Severely limited (1)	Somewhat limited (2)	Same (3)	Somewhat enhanced (4)	Greatly enhanced (5)
History-taking (1)	○	○	○	○	○	○
Physical examination (2)	○	○	○	○	○	○
Differential diagnosis (3)	○	○	○	○	○	○
Case write-ups (4)	○	○	○	○	○	○
Oral presentation (5)	○	○	○	○	○	○
Recommending/interpreting tests (6)	○	○	○	○	○	○
Forming clinical questions and retrieving evidence (7)	○	○	○	○	○	○
Collaborating as member of interprofessional team (8)	○	○	○	○	○	○

Display This Question:

If In which phase of the curriculum are you currently enrolled? = Post-PCE student

And In which phase of the curriculum are you currently enrolled? = Graduating student

Q30 Thinking about your virtual clinical sessions, how do you think these sessions have **limited or enhanced** development of your clinical skills.

	Not Applicable (6)	Severely limited (1)	Somewhat limited (2)	Same (3)	Somewhat enhanced (4)	Greatly enhanced (5)
History-taking (1)	o	o	o	o	o	o
Physical examination (2)	o	o	o	o	o	o
Differential diagnosis (3)	o	o	o	o	o	o
Case write-ups (4)	o	o	o	o	o	o
Oral presentation (5)	o	o	o	o	o	o
Recommending/interpreting tests (6)	o	o	o	o	o	o
Forming clinical questions and retrieving evidence (7)	o	o	o	o	o	o
Collaborating as member of interprofessional team (8)	o	o	o	o	o	o
Entering/Discussing orders (9)	o	o	o	o	o	o
Giving/Receiving patient handover (10)	o	o	o	o	o	o
Recognize patient requiring urgent/emergency evaluation (11)	o	o	o	o	o	o
Obtaining informed consent (12)	o	o	o	o	o	o
Performing general procedures (13)	o	o	o	o	o	o
Identifying system failures and contributing to a culture of safety and improvement (14)	o	o	o	o	o	o

Q56 **Thinking specifically about the clinical environment**, in what way has the transition to virtual learning influenced your relationships with...

	Much more negative (1)	Somewhat more negative (2)	Same (3)	Somewhat more positive (4)	Much more positive (5)
Faculty (1)	○	○	○	○	○
Residents (2)	○	○	○	○	○
Peers (3)	○	○	○	○	○

Q57 Compared to in-person interactions, how has the virtual format limited or enhanced faculty's ability to **assess** your competencies in...

	Not Applicable (1)	Severely limited (2)	Somewhat limited (3)	Same (4)	Somewhat enhanced (5)	Greatly enhanced (6)
History-taking (1)	○	○	○	○	○	○
Physical examination (2)	○	○	○	○	○	○
Differential diagnosis (3)	○	○	○	○	○	○
Case write-ups (4)	○	○	○	○	○	○
Oral presentation (5)	○	○	○	○	○	○
Recommending/interpreting tests (6)	○	○	○	○	○	○
Forming clinical questions and retrieving evidence (7)	○	○	○	○	○	○
Collaborating as a member of interprofessional team (8)	○	○	○	○	○	○

Q31 Given the transition away from in-person clinical experiences, how concerned are you about the following?

	Not at all concerned (1)	A little concerned (2)	Neutral (5)	Somewhat concerned (3)	Very concerned (4)
Gaining clinical experience overall (1)	○	○	○	○	○
Discerning my interest in a specialty (2)	○	○	○	○	○
Working as part of a clinical team (3)	○	○	○	○	○
Obtaining letters of recommendation (4)	○	○	○	○	○
Learning about the culture of a specialty (5)	○	○	○	○	○
Opportunity to rotate at a specific hospital site (6)	○	○	○	○	○
Observing other health professions (7)	○	○	○	○	○
Practicing communication skills with patients (8)	○	○	○	○	○
Other (9)	○	○	○	○	○

Q32 As part of your virtual clinical experiences, did you use Aquifer case modules?

- ○ Yes (1)
- ○ No (2)

Skip To: Q34 If As part of your virtual clinical experiences, did you use Aquifer case modules? = No

Q33 In comparison to the in person experiences that virtual learning is replacing, how have Aquifer modules **limited or enhanced** your clinical skills?

	Not applicable (6)	Severely limited (1)	Somewhat limited (2)	Same (3)	Somewhat enhanced (4)	Greatly enhanced (5)
History-taking (1)	○	○	○	○	○	○
Physical examination (2)	○	○	○	○	○	○
Differential diagnosis (3)	○	○	○	○	○	○
Recommending/interpreting tests (4)	○	○	○	○	○	○
Case write-ups (5)	○	○	○	○	○	○
Oral presentation (6)	○	○	○	○	○	○

Q34 As part of your virtual clinical experiences, have you had a Telehealth visit in one of your virtual clinic sessions?

- ○ Yes (1)
- ○ No (2)

Skip To: Q46 If As part of your virtual clinical experiences, have you had a Telehealth visit in one of your virt... = No

Q50 In comparison to the in person experiences that virtual learning is replacing, how have Telehealth visits **limited or enhanced** your clinical skills?

	Not Applicable (1)	Severely Limited (2)	Somewhat Limited (3)	Same (4)	Somewhat Enhanced (5)	Greatly Enhanced (6)
History Taking (1)	○	○	○	○	○	○
Physical Exam (2)	○	○	○	○	○	○
Differential Diagnosis (3)	○	○	○	○	○	○
Case Write Ups (4)	○	○	○	○	○	○
Oral Presentation (5)	○	○	○	○	○	○
Recommending/Interpreting tests (6)	○	○	○	○	○	○
Forming clinical questions and Retrieving evidence (7)	○	○	○	○	○	○
Collaborating as member of interprofessional team (8)	○	○	○	○	○	○

Q46 What unique opportunities do you see in virtual experiences for enhancing clinical training?

____

____

____

____

____

Q47 What are the biggest barriers that keep virtual experiences from complementing or enhancing clinical training?

____

____

____

____

____

Q49 **Thinking about technical aspects of virtual teaching that apply to all phases of the curriculum**

Q12 Which strategies do you find most effective to encourage student participation during virtual learning sessions?

	Did not use (6)	Very ineffective (1)	Somewhat ineffective (2)	Neutral (3)	Somewhat effective (4)	Very effective (5)
Chat function (1)	○	○	○	○	○	○
Breakout rooms (2)	○	○	○	○	○	○
Polling feature (3)	○	○	○	○	○	○
Hand raising (4)	○	○	○	○	○	○
Yes, No, Speed Up, Slow Down features (5)	○	○	○	○	○	○
Clap, Thumbs Up feature (6)	○	○	○	○	○	○
Annotation of slides as a group (7)	○	○	○	○	○	○
Cold Calling (8)	○	○	○	○	○	○

Q45 On a virtual learning platform, what class size do you find most conducive for your learning in a **large group?**

◯ <50 people (1)

◯ 50 to   (2)

◯ 100 to   (3)

◯ 150 to   (4)

◯ No preference (5)

Q8 On a virtual learning platform, what group size do you find most conducive for your learning?

	2 to 5 people (1)	6 to 10 people (2)	11 to 15 people (3)	16 to 20 people (4)	No preference (5)
Small group session (1)	◯	◯	◯	◯	◯
Break-out groups embedded in large-group sessions (2)	◯	◯	◯	◯	◯

Q9 What do you consider to be best practice for using the video function during virtual sessions (under the assumption that nobody struggles with connectivity)?

	Always turning on video function (1)	Turn on video when speaking (2)	Video always off (3)	No preference (4)
In a small group or break-out group (1)	◯	◯	◯	◯
In a large-group session (2)	◯	◯	◯	◯

Q14 Over the last few weeks, how often has your learning been negatively impacted by any of the following?

	Always (1)	Often (2)	Sometimes (3)	Rarely (4)	Never (5)
Unstable internet connection (1)	○	○	○	○	○
Software/Hardware issues (2)	○	○	○	○	○
Finding a quiet space (3)	○	○	○	○	○
Other (4)	○	○	○	○	○

Q24 Some courses record virtual sessions and make recordings available for later review. Please indicate how often you use recordings for any of the following:
(NOTE: this question is about class sessions, NOT recorded seminars or other resources assigned as prep)

	Every day (1)	Once or twice a week (2)	A few times per month (3)	Never (4)
To catch up on live sessions that I missed (1)	○	○	○	○
To go back and review class content (2)	○	○	○	○
Other (3)	○	○	○	○

Q60 HMS is committed to individual assessment of each student. How can we best accomplish this online?

____

Q40 HMS is in the process of developing an honor code for learning remotely. What expectations and behaviors do you expect of **yourself and your peers** to make this new virtual environment work for everyone?

____

____

____

____

____

End of Block: Default Question Block

Start of Block: Block 1
