# Supplementary material for: A hierarchy of needs for remote undergraduate medical education: lessons from the medical student experience
Source: BMC Med Educ. 2022 Jun 2;22:423. doi: 10.1186/s12909-022-03479-4 (PMC9161626; doi:10.1186/s12909-022-03479-4)
Supplement: Supplementary file 2 — Additional file 2. [file 12909_2022_3479_MOESM2_ESM.docx]

Additional file 2.

| **Theme** | **Included Codes** | **Definition of code** |
| --- | --- | --- |
| Sense of connection to people and course material through interpersonal interactions | Engage | Level of engagement, including engagement with coursework, attendance/participation |
|  | Camaraderie | Sense of camaraderie, including shared experience, sense of appreciation, bonding/community |
|  | Connected | Quality of interaction, including connectedness to others, knowing others well, feeling comfortable, closeness of relationships |
|  | Humanize | Humanizing of relationships |
|  | Interact | Amount of interactions, including contact with others, natural interactions, in-person interactions, proximity to others |
|  | Maintaining relationships | Comments specifically about maintaining existing relationships |
|  | New relationships | Comments specific to connecting with new faculty/peers, diversity of interactions |
|  | Engagement | Attendance, timeliness, participation, engagement, avoiding distractions |
|  | Collaboration | Collaboration with peers |
|  | Compassion | Compassion and understanding, recognizing competing demands |
|  | Faculty | Comments on interaction with faculty, i.e. feedback, office hours, longitudinal relationships |
|  | Small | Comments about learning in small groups |
|  | Student-faculty relationships | Encompasses faculty mentorship, coming up to faculty after class, learning from faculty |
|  | Student-student relationships | Encompasses learning from peers and social engagement |
|  | Group size | Comments about large vs. small group sizes working better for promoting learning |
|  | Patient interaction | Enjoying patient, real-case based learning |
|  | Participation | Encompasses engagement, asking questions |
|  | Attention | Comments about getting distracted, focus, staying engaged |
|  | Student-patient relationships | Includes interpersonal rapport with patients |
|  | Student-clinical team relationships | Comments about relationship between students and clinical team members |
|  | Physical environment | Comments about lack of in-person contact, interaction |
|  | Intellectual environment | Comments about the learning environment including hospital activities, rounds, lectures |
|  | Patient-centered communication | Comments about communicating with patients in a patient-centered manner |
|  | Interdisciplinary learning | Learning with others across disciplines, interdisciplinary teams |
| Role of technology and virtual resources for enhancement of learning | Virtual experience | Experience with virtual platform, comments specifically online/Zoom experience, comfort speaking up and asking questions |
|  | Resources | Use of external resources, closed-book exam, specific mention of unapproved actions |
|  | Technology | Use of technology for learning e.g., polls or visual aids, technical issues, faculty troubles/strengths with tech, pre-recorded |
|  | Creativity | Students, faculty, structure |
|  | Aquifer clinical reasoning | Use of Aquifer specifically for clinical reasoning development |
|  | History taking | Learning about taking a history virtually |
|  | Using technology for teaching/patient care | Integration of technology for the purposes of teaching and clinical care |
|  | History collection | Ability to collect history virtual |
|  | Clinical reasoning | Developing clinical reasoning skills in general |
| Effects of learning in an exclusively virtual environment | Learning | Comments about effects on learning |
|  | In-person | Same as in-person, no change |
|  | Learning | Comments about emphasizing learning over assessment/performance |
|  | Unable | Not able to assess, eliminate assessment student assessment in virtual environment |
|  | Alternative | New modality of assessment not included in other categories; self-assessment, completion of modules, written assignments/projects |
|  | Clinical | Simulations, observed telehealth visits, OSCE; observation-based |
|  | Length of class time | Encompasses breaks, length fatigue |
|  | Technology | Technical difficulties, internet challenges |
|  | Zoom | Comments about zoom and overall computer/virtual fatigue and disengagement |
|  | Physical Exam | Comments about physical exam (e.g. can’t do it, can’t learn skills) |
|  | Passive learning | Comments about passive learning primarily via Aquifer cases |
| Professionalism in the virtual environment | Cheating | Explicit mention of cheating without operational example/action |
|  | Prep | Coming prepared for class |
|  | Etiquette | Virtual-specific etiquette, i.e. video, muting mic, screenshots |
|  | Trust | Trust students, honor code not necessary |
|  | Integrity | Explicit mention of integrity/honesty or related terms without operational example/action |
|  | Expectations | Clear expectations, team norms, and communication |
|  | Accountability | Structure of class, accountability to projects |
|  | Difficulty with professional discernment | Discerning between specialties, culture, inpatient vs. outpatient |
|  | Independence | Comments about having less independence |
|  | Telehealth | Familiarity with telehealth and opportunities to engage with telehealth |
|  | Social determinants of health | Introduction to social determinants of health, including home assessments, rural health |
| Flexibility | Independent (asynchronous) learning | Encompasses flexibility with class timing, watching videos/learning on own, or alternatively, interest in synchronous learning |
|  | Time efficiency, flexibility | Comments about time efficiency and flexibility in virtual environment |
|  | Fostering self-directed learning | Self-directed learning, self-study in the virtual environment |
| Adaptation of assessment and feedback to virtual environment | Written | Written exams as means of virtual assessment |
|  | Oral exam | Oral exams as means of virtual assessment |
|  | Presentation | Presentations as means of virtual assessment |
|  | Assessments | Comments about assessment in the virtual environment |
|  | Lack of real-time processing | Comments about ability to process information and feedback in real-time |
|  | Faculty feedback | Comments about receiving faculty feedback in virtual courses |
