# Supplementary material for: A hierarchy of needs for remote undergraduate medical education: lessons from the medical student experience
Source: BMC Med Educ. 2022 Jun 2;22:423. doi: 10.1186/s12909-022-03479-4 (PMC9161626; doi:10.1186/s12909-022-03479-4)
Supplement: Supplementary file 3 — Additional file 3. [file 12909_2022_3479_MOESM3_ESM.docx]

**Additional file 3.**

*Likert response options were collapsed into categories and analyzed using the Chi-Square Test of Independence. In the few instances where means are analyzed, Independent Samples t-Tests were employed.*

**1. Effects of Relationships on ability to learn and participate in class**

**1.1 Relationships with peers more negative**

Most respondents (59%) report that virtual learning has damaged their relationships with peers. This group is more likely that the group that reports their relationship with peers is about the same or more positive to say that virtual learning has limited their ability to learn.

**% Limited My Ability to Learn**

|  | Relationship with Peers more Negative  (59% n=106) | Relationship with Peers the Same or More Positive  (41% n=74) | Chi-Square p-Value |
| --- | --- | --- | --- |
| Small group discussion | 74 | 58 | .020 |
| CBCL sessions | 77 | 74 | .747 |
| Lectures/Seminars | 59 | 40 | .013 |
| Office hours | 64 | 42 | .021 |
| Review sessions | 59 | 38 | .019 |
| Student presentations | 51 | 41 | .274 |

**% Less Comfortable Participating**

|  | Relationship with Peers More Negative (59% n=106) | Relationship with Peers Same/More Positive (41% n=74) | Chi-Square p-Value |
| --- | --- | --- | --- |
| Ask a question | 63 | 48 | .146 |
| Answer a question | 64 | 42 | .016 |
| Challenge ideas | 62 | 54 | .567 |
| Make a presentation | 35 | 19 | .087 |

**1.2 Relationships with faculty are more negative**

Most respondents (55%) report that virtual learning has damaged their relationships with faculty. This group is more likely than the group that reports their relationship with faculty is about the same or more positive to say that virtual learning has limited their ability to learn.

**% Limited My Ability to Learn**

|  | Relationship with Faculty more Negative  (55% n=99) | Relationship with Faculty the Same or More Positive  (45% n=81) | Chi-Square p-Value |
| --- | --- | --- | --- |
| Small group discussion | 75 | 58 | .021 |
| CBCL sessions | 78 | 71 | .429 |
| Lectures/Seminars | 62 | 37 | .001 |
| Office hours | 69 | 40 | .003 |
| Review sessions | 59 | 40 | .031 |
| Student presentations | 67 | 27 | <.001 |

**% Less Comfortable Participating**

|  | Relationship with Faculty More Negative (55% n=99) | Relationship with Faculty Same/More Positive (45% n=81) | Chi-Square p-Value |
| --- | --- | --- | --- |
| Ask a question | 67 | 43 | <.001 |
| Answer a question | 67 | 39 | <.001 |
| Challenge ideas | 71 | 44 | <.001 |
| Make a presentation | 44 | 11 | <.001 |

**2. Effects of environment (unstable internet/access to quiet space) on ability to learn and participate in class**

**2.1 Problems finding a quiet space**

**% Limited My Ability to Learn**

|  | Rarely/Never problems quiet space  (47%, n =83) | Sometimes/Often/Always problems quiet space  (53%, n =95) | Chi-Square p-Value |
| --- | --- | --- | --- |
| Small group discussions | 66 | 69 | .712 |
| CBCL sessions | 76 | 76 | .933 |
| Lectures/Seminars | 46 | 56 | .178 |
| Office hours | 55 | 56 | .904 |
| Review sessions | 45 | 55 | .259 |
| Student presentations | 39 | **55** | .067 |

**% Less Comfortable Participating**

|  | Rarely/Never problems quiet space  (47%, n =83) | Sometimes/Often/Always problems quiet space  (53%, n =95) | Chi-Square p-Value |
| --- | --- | --- | --- |
| Ask a question | 54 | 59 | .495 |
| Answer a question | 51 | 59 | .283 |
| Challenge ideas | 56 | 63 | .410 |
| Make a presentation | 25 | 30 | .498 |

**2.2 Problems with unstable internet**

**% Limited My Ability to Learn**

|  | Rarely/Never unstable internet  (39%, n =70) | Sometimes/Often/Always unstable internet  (61%, n =108) | Chi-Square p-Value |
| --- | --- | --- | --- |
| Small group discussions | 64 | 69 | .492 |
| CBCL sessions | 76 | 76 | .933 |
| Lectures/Seminars | 41 | **58** | **.035** |
| Office hours | 55 | 55 | .997 |
| Review sessions | 45 | 54 | .331 |
| Student presentations | 34 | **55** | **.026** |

**% Less Comfortable Participating**

|  | Rarely/Never unstable internet  (39%, n =70) | Sometimes/Often/Always unstable internet  (61%, n =108) | Chi-Square p-Value |
| --- | --- | --- | --- |
| Ask a question | 61 | 54 | .391 |
| Answer a question | 52 | 57 | .565 |
| Challenge ideas | 61 | 59 | .818 |
| Make a presentation | 26 | 29 | .688 |

**2.3 Problems with BOTH unstable internet and quiet space**

43% (n=76) reported experiencing issues with *both* internet connection and finding quiet space at least sometimes. Despite these challenges there was little effect on the students’ ability to learn or participate.

**% Limited My Ability to Learn**

|  | Unstable internet and trouble finding quite space at least sometimes  (43%, n =76) | Other  (57%, n =102) | Chi-Square p-Value |
| --- | --- | --- | --- |
| Small group discussions | 69 | 66 | .765 |
| CBCL sessions | 78 | 75 | .705 |
| Lectures/Seminars | 57 | 47 | .156 |
| Office hours | 57 | 53 | .682 |
| Review sessions | 56 | 46 | .255 |
| Student presentations | 60 | 36 | .005 |

**% Less comfortable participating**

|  | Unstable internet and trouble finding quite space at least sometimes  (43%, n =76) | Other  (57%, n =102) | Chi-Square p-Value |
| --- | --- | --- | --- |
| Ask a question | 58 | 56 | .885 |
| Answer a question | 60 | 52 | .295 |
| Challenge ideas | 59 | 60 | .934 |
| Make a presentation | 27 | 29 | .787 |

There is little relationship between environmental issues and preference for ideal time allocation. Examination of both means and distributions across the rarely/never and often/always categories shows little difference (and no statistically significant differences) between the 2 groups.

**Ideal time allocation**

|  | Unstable internet and trouble finding quite space at least sometimes  (43%, n =76) | Other  (57%, n =102) | Independent Sample T p-Value |
| --- | --- | --- | --- |
| Mean Hours Virtual Class | 2.51 | 2.34 | .336 |
| Mean Hours Work Remotely by Oneself | 3.28 | 2.95 | .091 |
| Mean Hours Collaborate Remotely with Peers | 1.72 | 1.53 | .109 |

Unstable internet and trouble finding quite space did *not* affect relationships.

**Relationships**

|  | Unstable internet and trouble finding quite space at least sometimes  (43%, n =76) | Other  (57%, n =102) | Chi-Square p-Value |
| --- | --- | --- | --- |
| Relationship with Faculty and Peers more Negative  (49% n=79) | 52 | 48 | .580 |
| Other  (51% n=81) | 48 | 52 |  |
